# Effects of *Lagenaria siceraria (Molina) Standl* polysaccharides on growth performance, immune function, cecum microorganisms and short-chain fatty acids in broilers

**DOI:** 10.3389/fvets.2024.1428623

**Published:** 2024-10-15

**Authors:** Zhenping Zhang, Shiqi Dong, Jinrong Li, Maimaiti Aizezi, Peng Huang, Saifuding Abula, Zhanhai Mai, Dandan Liu, Adelijiang Wusiman

**Affiliations:** ^1^Xinjiang Key Laboratory of New Drug Study and Creation for Herbivorous Animals (XJ-KLNDSCHA), College of Veterinary Medicine, Xinjiang Agricultural University, Urumqi, China; ^2^College of Veterinary Medicine, Southwest University, Chongqing, China

**Keywords:** growth performance, immune function, intestinal microbiota, *Lagenaria siceraria (Molina) Standl* polysaccharides, short-chain fatty acids

## Abstract

In this study, *Lagenaria siceraria (Molina) Standl* polysaccharides (LSP) was prepared using the water-alcohol precipitation method to evaluate its effects on growth performance, slaughter performance, cytokines, immune organ indices, cecal short-chain fatty acids (SCFAs), and microbial community structure in broiler chickens when added to the basal diet. Seventy-five broiler chickens were selected and randomly divided into five groups, with 15 chickens per group. All groups were fed a basal diet for 7 days. From 7 days of age, the control group continued to receive the basal diet, while the positive drug group was fed a diet supplemented with Astragalus polysaccharides (APS, 100 g/kg) in addition to the basal diet. The experimental groups were fed diets containing different concentrations of LSP (50, 100, and 200 g/kg) in addition to the basal diet, and the supplementation continued for 42 days. The findings indicated that the incorporation of LSP into the feed significantly enhanced average daily weight gain (ADWG), average daily feed intake (ADFI), feed to gain ratio (F/G), dressing percentage, percentage of breast muscle, percentage of leg muscle, and percentage of abdominal fat while concurrently reducing drip loss rate and cooking loss rate (*p* < 0.01) in comparison to the control group. Additionally, it significantly augmented the levels of interleukin-4 (IL-4) and interleukin-12 (IL-12) in cytokines, secreted immunoglobulin A (SIgA) and immunoglobulin G (IgG) in immunoglobulins, as well as immune organ indicators (*p* < 0.05). Furthermore, LSP also modulated the intestinal microbiome composition by increasing the abundance of *Bacteroides* species and significantly changing concentrations of specific short-chain fatty acids (SCFAs) such as propionic acid, isobutyric acid, acetic acid, and isovaleric acid (*p* < 0.01). These results suggest that dietary supplementation with LSP can effectively regulate intestinal microbiome composition while promoting short-chain fatty acid production. The alterations in microbial characteristics ultimately contribute to improved intestinal immunity and immune organ development as well as enhanced production performance and immune function in broilers.

## 1 Introduction

Intestinal health is a prerequisite for the optimal growth and development of livestock and poultry. The intestinal well-being of these animals is determined by factors such as the composition of their gut microbiota, host immunity (including mucosal barrier function), nutritional intake, and environmental conditions (1). Issues related to intestinal health in livestock and poultry farming often arise from dysbiosis within the gut microbiota, impairment of the mucosal barrier integrity, or inflammatory responses (2). Although antibiotics, vaccines, chemical drugs, and immune stimulants have been utilized in farming with significant effectiveness, their excessive use has resulted in numerous side effects (3). For instance, overuse of antibiotics in farming can contribute to antibiotic resistance development, disrupt the microbial environment, and cause drug residues that contaminate the environment (4). Therefore, there is an urgent requirement for a novel feed additive that possesses safety, efficiency, absence of residues, and immune-enhancing properties to ensure the robust development of the breeding industry.

*Lagenaria siceraria*, belonging to the Cucurbitaceae family, is a plant with both medicinal and nutritional value (5). In traditional medicine, it has been widely used to treat various diseases. The pulp of the *Lagenaria siceraria* can serve as both an emetic and a laxative, and it also has diuretic properties (6). The vines and tendrils of the plant, which share similar medicinal properties with the flowers, can be used to treat skin conditions like leprosy. The seeds are bitter, cold in nature, and toxic, and they can be used to treat dental diseases, facial swelling, and urinary retention (7). Additionally, *Lagenaria siceraria* is rich in nutrients, containing polysaccharides, proteins, various trace elements, vitamin C, and trypsin inhibitors. These components help enhance immunity, promote antibody synthesis, and improve disease resistance in animals (8).

Polysaccharides derived from natural plants exhibit anti-tumor, antioxidant, anti-inflammatory, hypoglycemic, blood pressure-lowering, immune-regulating, properties, among others. Simultaneously, they possess the advantages of non-toxicity, absence of residue in animal bodies and products, and resistance to occurrence. These attributes play a pivotal role in promoting the sustainable development of modern animal husbandry (9). As a significant active ingredient in *Lagenaria siceraria*, *Lagenaria siceraria (Molina) Standl* polysaccharide (LSP) has progressively garnered attention from researchers. Studies have revealed that LSP hold potential for treating jaundice, diabetes, ulcers, hemorrhoids while also alleviating symptoms associated with colitis (10). Furthermore it has been found to possess antipsychotic and antihypertensive properties along with its ability to alleviate congestive heart failure and skin diseases (11). The molecular weight of LSP was determined to be 78 kDa by Kaushik Ghosh et al., who identified structural fragments such as 1–4 linked *α*-D-galacturonic acid, 1–2 linked 3-O-acetylmethyl-α-D-galacturonic acid and 1–4 linked *β*-D-galactose within the polysaccharide structure (12). Zhou et al. demonstrated that LSP50 exhibits immunomodulatory effects on immune organ indices, H9N2-specific IgG levels, cytokine profiles (including IFN-*γ*, IL-2, IL-4, and IL-5), and the CD3eCD8a T cell ratio. Furthermore, sequencing analysis revealed that LSP50 regulates PLA2G12B and PTGDS genes involved in arachidonic acid pathway to modulate immune response (13). Wusiman et al. (14) used polysaccharide extracts as vaccine adjuvants for chicken immunity. It was found that the administration of LSP led to an increase in HI titers, enhanced production of antigen-specific IgG NDV antibodies, increased proliferation of splenic lymphocytes, and elevated immune organ indices (14).

Therefore, the objective of this study is to assess the potential of LSP as a natural dietary supplement for enhancing chicken health, with a specific focus on its impact on growth performance, slaughter performance, immune function, gut microbiota composition, and short-chain fatty acid concentration. The effects of LSP on chicken growth and slaughter performance were evaluated by monitoring changes in body weight, feed consumption, carcass weight, and net body weight. Furthermore, the influence of LSP on intestinal functionality in chickens was analyzed by investigating its effects on intestinal morphology, immune function markers such as globulin content and immune organ index values, short-chain fatty acid concentration levels, and gut microbial composition.

## 2 Materials and methods

### 2.1 Preparation and structural analysis of LSP

The crude polysaccharides were extracted using water decoction and ethanol precipitation methods, followed by protein removal through the Sevag method. Subsequently, the crude polysaccharides underwent a process of separation and purification involving elution with deionized water. The elution fractions were collected, merged, and then subjected to freeze-drying to obtain a pure and homogeneous form of LSP (14). The monosaccharide composition of LSP was analyzed using Fourier-transform infrared spectroscopy and high-performance liquid chromatography techniques.

### 2.2 Animal grouping and immunization procedures

A total of 75 newly hatched Sanhuang broilers, aged 0 days, were randomly allocated into five groups with 15 broilers per group after a seven-day period of domestication. The control group was fed a basal diet daily (Tiankang Biotech Co., Ltd.). The LSP treatment group was further divided into three subgroups, each receiving different doses of LSP in the basal diet: LSP-low (LSP-l, 50 g/kg), LSP-mid (LSP-m, 100 g/kg), and LSP-high (LSP-h, 200 g/kg). The positive drug group received a basal diet supplemented with 100 g/kg of APS (Sichuan Dingjian Animal Pharmaceutical Co., Ltd.). At day 8, the broilers were intranasally immunized with a commercially available inactivated H9N2 vaccine (Urumqi Center for Disease control and Prevention) followed by a secondary immunization at day 22. The control group received intranasal administration of physiological saline solution. Dissection was performed at day 50 with *ad libitum* feeding throughout the experimental period. Animal welfare guidelines for experimental animals were strictly adhered to during the study (Table 1).

**Table 1:** Basic feed ingredients and nutrient levels (Dry basis).

Ingredient	1–4 Weeks (%)	5–9 Weeks (%)	Nutrient level	1–4 Weeks (MJ/kg)	5–9 Weeks (MJ/kg)
Corn	62.30	67.20	Metabolizable Energy (MJ/kg)	12.96	13.89
Soybean meal	23.70	19.70	Crude Protein (%)	20.00	18.50
Cottonseed meal	4.73	5.26	Lysine (%)	1.00	0.80
Soybean oil	3.26	3.71	Methionine (%)	0.40	0.30
Limestone	1.27	1.18	Methionine + Cysteine (%)	0.76	0.84
Corn gluten meal	2.23	3.12	Calcium (%)	0.90	0.90
Dicalcium phosphate	1.76	1.65	Available Phosphorus (%)	0.40	0.30
Salt	0.32	0.30			
L-Lysine	0.25	0.21			
DL-Methionine	0.10	0.10			
Choline Chloride	0.08	0.05			

Vitamin A 2700 IU, Vitamin D 2000 IU, Vitamin E 10 IU, Vitamin K 0.5 mg, Vitamin B12 0.015 mg, Riboflavin 6 mg, Pantothenic Acid 10 mg, Niacin 40 mg, Pyridoxine 3 mg, Biotin 0.15 mg, Choline 300 mg, Iron 50 mg, Copper 4 mg, Manganese 50 mg, Zinc 40 mg, Iodine 0.35 mg, Selenium 0.18 mg.

### 2.3 Sample collection

At 50 days of age, blood samples were collected from the broilers’ hearts and centrifuged at 3000 rpm for 15 min. The resulting serum was stored at −80°C. Following ether inhalation anesthesia, the broilers were euthanized by cervical vertebrae dislocation. The immune organs including thymus, spleen, bursa of fabricius, cecal tonsils, and jejunum were harvested to calculate the immune organ index. [immune organ index = organ weight (g) / body weight (kg)]. A portion of these tissues underwent fixation in a paraformaldehyde solution for histological observations while another portion was stored at −80°C for future use (15).

### 2.4 Measurement of growth performance

The broiler chickens were weighed at 8 and 50 days, following a 12-h fasting period, in order to determine the average daily feed intake (ADFI), average daily weight gain (ADWG), and feed to gain ratio (F/G) (16).

### 2.5 Determination of slaughtering performance

The following parameters were calculated at 50 days: dressing percentage, percentage of half-eviscerated yield, percentage of eviscerated yield, percentage of breast muscle, percentage of leg muscle, percentage of abdominal fat, drip loss rate, and cooking loss rate (17).

### 2.6 Determination of immunoglobulins

Establishment of ELISA Method: 1.59 g of sodium carbonate and 2.93 g of sodium bicarbonate were weighed into a beaker and dissolved in 800 mL of distilled water. The pH was adjusted to 9.6 using sodium hydroxide, and then water was added to make up to 1,000 mL, preparing a carbonate buffer solution (PBS) for later use. The H9N2 avian influenza antigen was diluted in PBS with a pH of 9.6 and coated on the ELISA plate overnight at 4°C. After washing the plate with PBST, the ELISA plate was blocked with a 1% bovine serum albumin solution at 37°C for 2 h. The diluted test serum or intestinal juice was added and incubated for 1 h, followed by adding HRP-labeled IgG or IgA antibody and incubating for another hour. Color development was achieved using TMB substrate solution in the dark, and finally terminated by adding 2 M H_2_SO_4_ before measuring absorbance at A450 (14).

### 2.7 Cytokine assay

Serum samples were collected and stored at −80°C until analysis. Prior to the assay, samples were thawed at room temperature and diluted as necessary. In accordance with the manufacturer’s instructions, commercially available enzyme-linked immunosorbent assay (ELISA) kits (FanKew, Shanghai, China) were utilized to quantify the concentrations of Interleukin-4, Interleukin-5, Interleukin-12, and Interferon-gamma (IL-4, IL-5, IL-12, and IFN-*γ*) in serum samples.

### 2.8 Histological change detection

The thymus, spleen, fabricius, cecal tonsils, and jejunum were immersed in a 4% paraformaldehyde fixative for fixation purposes. Subsequently, the tissues underwent histological processing to obtain sections that were stained using the hematoxylin and eosin (HE) method for observing alterations in their histological morphology (14).

### 2.9 Microbial diversity of the cecum and determination of SCFAs

On the 50th day, fecal samples were collected from the control group, APS group, and LSP group receiving the most effective treatment and stored in liquid nitrogen tanks for subsequent transportation to the laboratory for DNA extraction. The DNA concentration and quality were evaluated using a spectrophotometer. The V3-V4 region of bacterial 16S rRNA gene was amplified by PCR using universal primers, followed by purification. Subsequently, a sequencing library was established and accurate quantification was performed using a Qubit fluorometer. PANOMIX Biotech conducted computer sequencing analysis on gut microbiota and SCFA-related indicators.

### 2.10 Statistical analysis

The growth performance, slaughter performance, cytokines, and SCFAs concentrations in cecal digesta were subjected to one-way analysis of variance (ANOVA) using SPSS 24.0 statistical software. To assess significant differences among treatment means, Duncan’s multiple range test was utilized. All reported findings represent the mean values accompanied by standard errors of the mean (SEM). Graphs were created employing GraphPad Prism 5 and Origin 2022 software.

## 3 Results

### 3.1 Composition and structure of LSP

**Figure 1:**
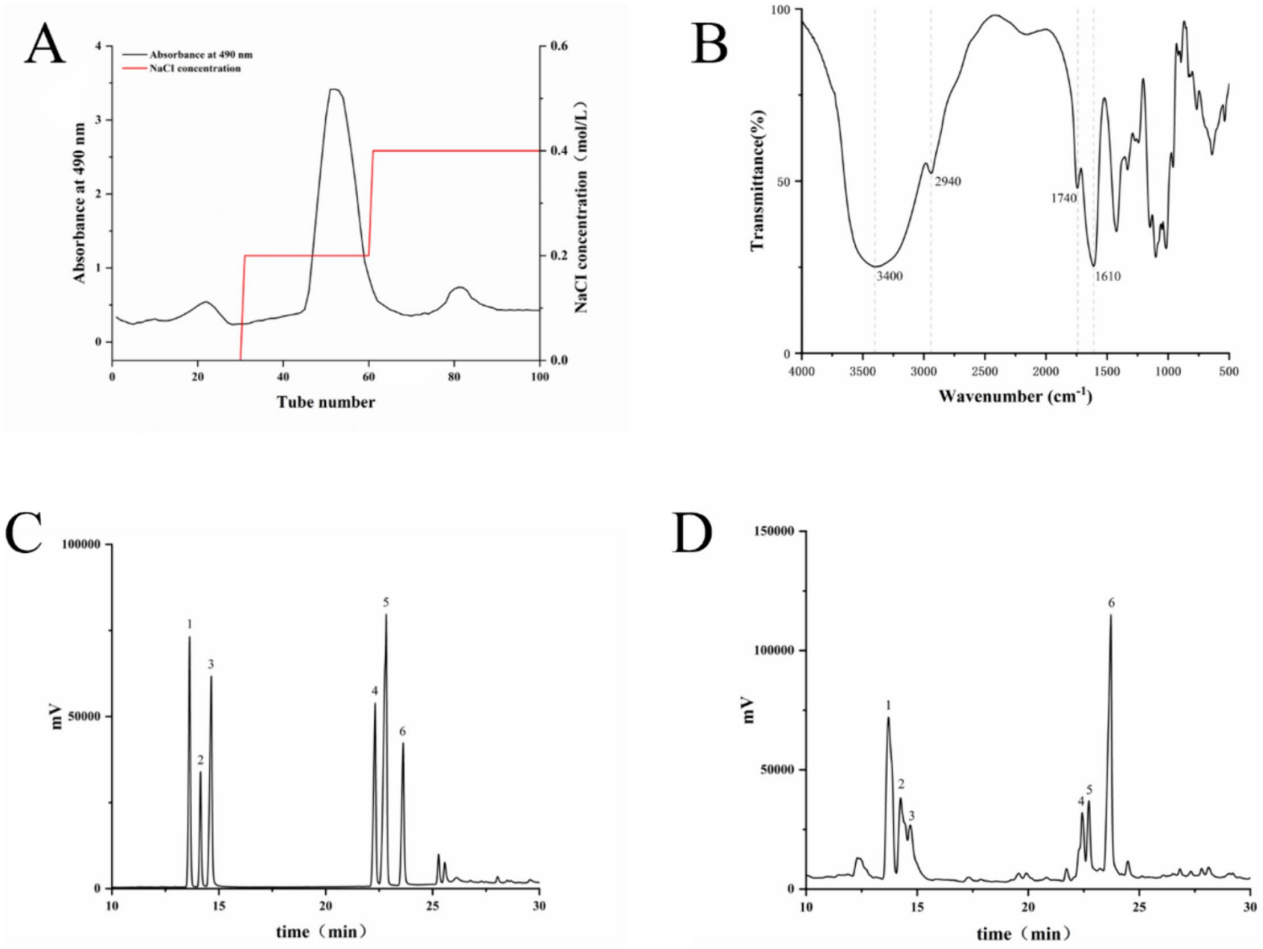
Composition and structural analysis of LSP **(A)** Elution curve of LSP, **(B)** Fourier-transform infrared spectroscopy (FT-IR) analysis of LSP, **(C)** Mixed standard, **(D)** Monosaccharide composition of LSP. Numbers 1–6 represent xylose, arabinose, mannose, galactose, glucose, and rhamnose, respectively.

The total LSP was obtained through water extraction and ethanol precipitation, followed by purification using a DEAE column and elution with NaCl to isolate the main polysaccharide fractions (Figure 1). The HPLC analysis revealed that the composition of LSP consisted of galactose (28.60%), rhamnose (28.83%), arabinose (15.76%), xylose (11.11%), mannose (8.55%) and glucose (7.16%). Rhamnose and galactose were identified as the predominant monosaccharides in LSP, collectively accounting for 57.43% of the total monosaccharide content (Table 1).

The infrared spectral analysis results are presented in Figure 1D. The intense absorption peak at 3440 cm^−1^ is attributed to the stretching vibration of O-H, while the absorption peak at 2940 cm^−1^ corresponds to C-H stretching vibration (18). The presence of carbonyl groups in the polysaccharide is suggested by an absorption peak around 1740 cm^−1^, and the C-O stretching causes an absorption peak near 1,630 cm^−1^. Peaks between 1,200 and 1,000 cm^−1^ represent the stretching vibration of C-O-C bonds, confirming the presence of glucuronic acid and adjacent acetyl groups (19).

### 3.2 Effect of LSP on broiler growth performance

The impact of LSP on the growth performance of broilers is presented in Table 2. The average daily weight gain (ADWG), average daily feed intake (ADFI), and feed to gain ratio (F/G) were significantly higher in the LSP-h, LSP-m, and LSP-l groups compared to the control group (*p* < 0.05). There was no significant difference observed among all groups regarding LSP levels (*p* > 0.05). Moreover, the F/G of the LSP-l group showed a more pronounced improvement, with no significant difference compared to the APS group (*p* > 0.05).

**Table 2:** Effect of LSP on growth performance of broilers.

Items			Groups				
	Control	APS	LSP-l	LSP-m	LSP-h	SEM	*p-*value
Average daily feed intake(g)	17.54^c^	26.43^a^	23.21^b^	22.68^b^	24.31^b^	0.83	< 0.01
Average daily weight gain(g)	103.00^c^	125.25^a^	114.00^b^	114.25^b^	117.50^ab^	4.13	< 0.01
Feed to gain ratio(g/g)	5.89^a^	4.74^c^	4.91^bc^	5.04^b^	4.84^bc^	0.10	< 0.01

Different lowercase letters on the data shoulder tags indicate significant differences (*P* < 0.05).

### 3.3 Effect of LSP on the slaughtering performance of broiler chickens

The impact of LSP on the slaughter performance of broiler chickens is demonstrated in Table 3. Compared to the control group, the inclusion of LSP in the feed significantly increased various parameters including dressing percentage, percentage of half-eviscerated yield, percentage of eviscerated yield, percentage of breast muscle, percentage of leg muscle, drip loss rate, and cooking loss rate (*p* < 0.01). Among them, the LSP-l group exhibited significantly greater improvements in the percentage of half-eviscerated yield, percentage of eviscerated yield, percentage of breast muscle, and percentage of leg muscle compared to the LSP-m and LSP-h groups (*p* < 0.01). When compared with the positive control drug APS group, there were no significant differences observed in dressing percentage, percentage of half-eviscerated yield, percentage of eviscerated yield, percentage of breast muscle, percentage of abdominal fat, and drip loss rate (*p* > 0.05).

**Table 3:** Effect of LSP on slaughter performance indexes of broiler chickens.

Items			Groups				
	Control	APS	LSP-l	LSP-m	LSP-h	SEM	*P*-value
Dressing percentage	93.94^b^	97.12^a^	96.44^a^	95.83^a^	96.98^a^	0.64	<0.01
Percentage of half-eviscerated yield	91.87^c^	94.77^a^	93.83^ab^	93.04^bc^	93.44^b^	0.59	< 0.01
Percentage of eviscerated yield	73.71^c^	77.73^a^	76.35^ab^	74.61^bc^	75.20^bc^	0.78	< 0.01
Percentage of breast muscle	11.15^d^	17.35^a^	15.87^ab^	13.97^c^	15.35^bc^	0.77	< 0.01
Percentage of leg muscle	11.74^c^	17.60^a^	15.55^b^	15.06^b^	15.51^b^	0.77	< 0.01
Percentage of abdominal fat	2.27^a^	1.43^b^	1.61^b^	1.69^b^	1.54^b^	0.12	< 0.01
Drip loss rate	2.58^a^	1.34^b^	1.56^b^	1.52^b^	1.56^b^	0.11	< 0.01
Cooking loss rate	45.01^a^	38.19^c^	41.59^b^	42.03^b^	40.63^b^	0.81	< 0.01

Different lowercase letters on the data shoulder tags indicate significant differences (*P* < 0.05).

### 3.4 Effects of LSP on immune function

**Figure 2:**
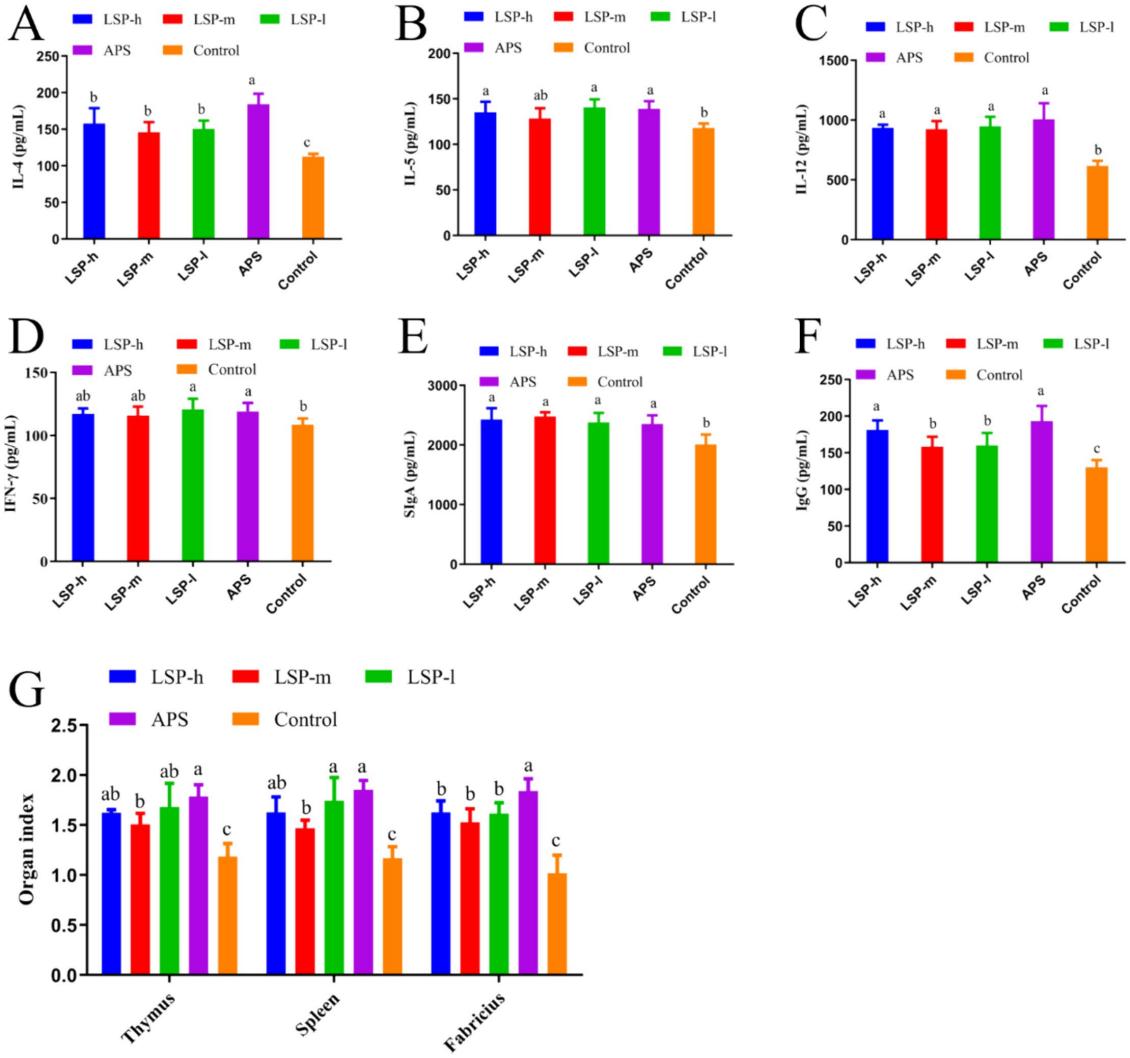
Repercussions of LSP on cytokine levels and immune organ indices. **(A)** IL-4 Content. **(B)** IL-5 Content. **(C)** IL-12 Content. **(D)** IFN-*γ* Content. **(E)** SIgA Content. **(F)** IgG Content. **(G)** Immunological organ index. Bars with different superscripts **(A–D)** indicate significant differences (*p* < 0.05).

The impact of LSP on cytokines, immunoglobulins, and immune organ indices is illustrated in Figure 2. In comparison to the control group, supplementation with LSP significantly stimulated the production of IL-4, IL-12, SIgA, and IgG. Among the different doses of LSP groups, there were no significant differences observed in the expression levels of other indexes except for IgG. The expressions of IL-12, IFN-*γ*, and SIgA did not exhibit significant differences compared to the APS group (*p* > 0.05). Furthermore, thymus, spleen, and bursa of fabricius indexes were significantly increased in the LSP group when compared to the control group (*p* < 0.05). Notably, this enhancement effect was more pronounced in the LSP-l group than in both the LSP-m and LSP-h groups; However it did not differ significantly from that observed in the APS group (*p* > 0.05).

### 3.5 HE stained section analysis

**Figure 3:**
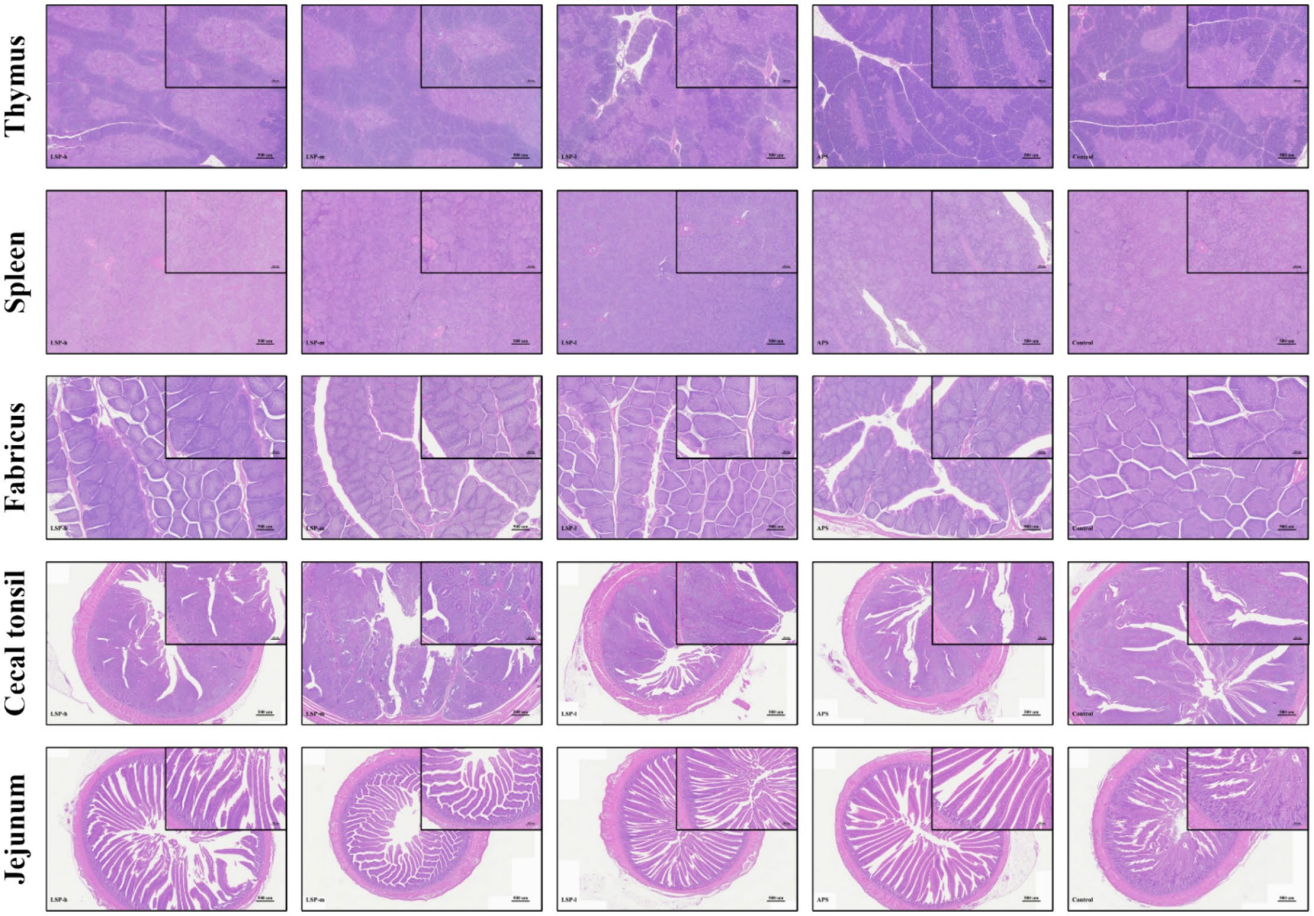
Tissue section analysis results.

To evaluate the impact of LSP on broiler chickens’ immune organs and intestines, we examined tissue changes in the thymus, spleen, fabricius, cecal tonsils, and ileum. The results depicted in Figure 3 demonstrate that HE staining observations revealed no discernible alterations in the structure of immune organs and intestinal tissues among all experimental groups of broiler chickens. All groups exhibited a normal physiological morphology.

### 3.6 The influence of LSP on intestinal microbial abundance

**Figure 4:**
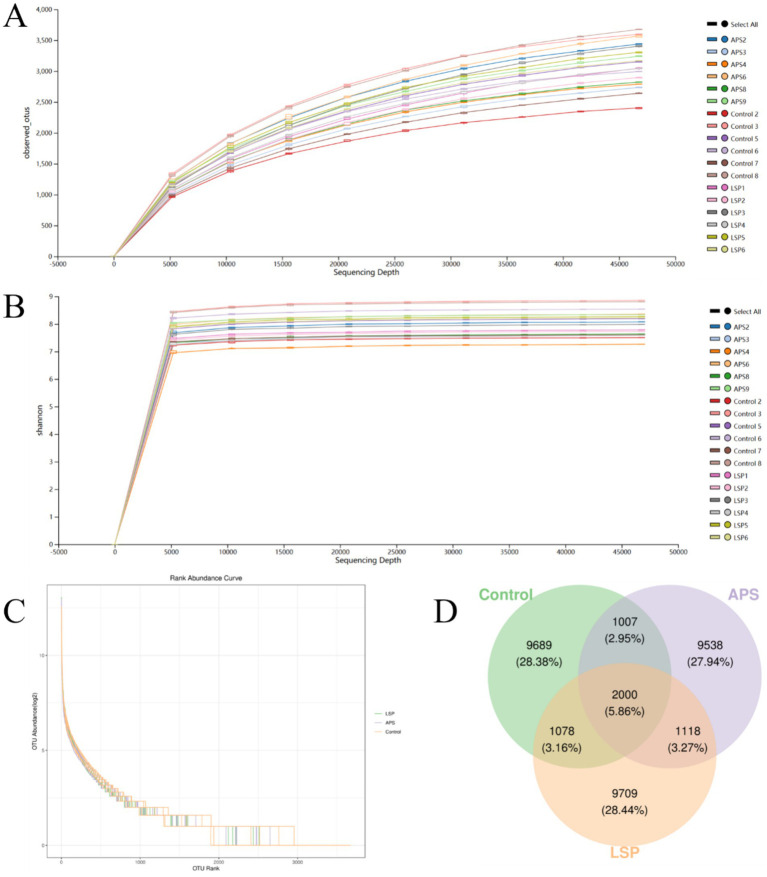
The influence of LSP on intestinal microbial abundance **(A)** Observed_species curve. **(B)** Shannon curve. **(C)** Rank abundance curve. **(D)** Venn diagram of ASVs/OTUs.

The impact of LSP on the abundance of intestinal microbiota in broiler chickens is depicted in Figure 4. To ensure accurate representation of microbial composition in cecal content, observed species curves and Shannon curves were employed to assess sequencing depth (20). The results indicated that a sequencing depth of 50,000 reads covered the majority of microbial species, and even with further sequencing, no new species were identified, ensuring the reliability of the data obtained through sequencing (Figures 4A,B). The Rank abundance curve illustrated the distribution pattern of high-abundance and rare ASV/OTU within the microbial community (21). The graph shows initial fluctuations in samples, but the curves stabilize after reaching a richness value of 3,000 (Figure 4C). This suggests a uniform overall distribution of microbial species in each sample, providing a robust foundation for further comprehensive analysis.

The Venn diagram in Figure 4D illustrates the composition of the intestinal microbial community, revealing that there were 9,709 OTUs for the LSP group, 9,538 for the APS group, and 9,689 for the control group. Among these groups, a total of 2000 OTUs were shared, with 1,078 OTUs shared between the LSP group and control group, and 1,007 OTUs shared between the APS group and control group.

### 3.7 The impact of LSP on the diversity of intestinal microbiota

**Figure 5:**
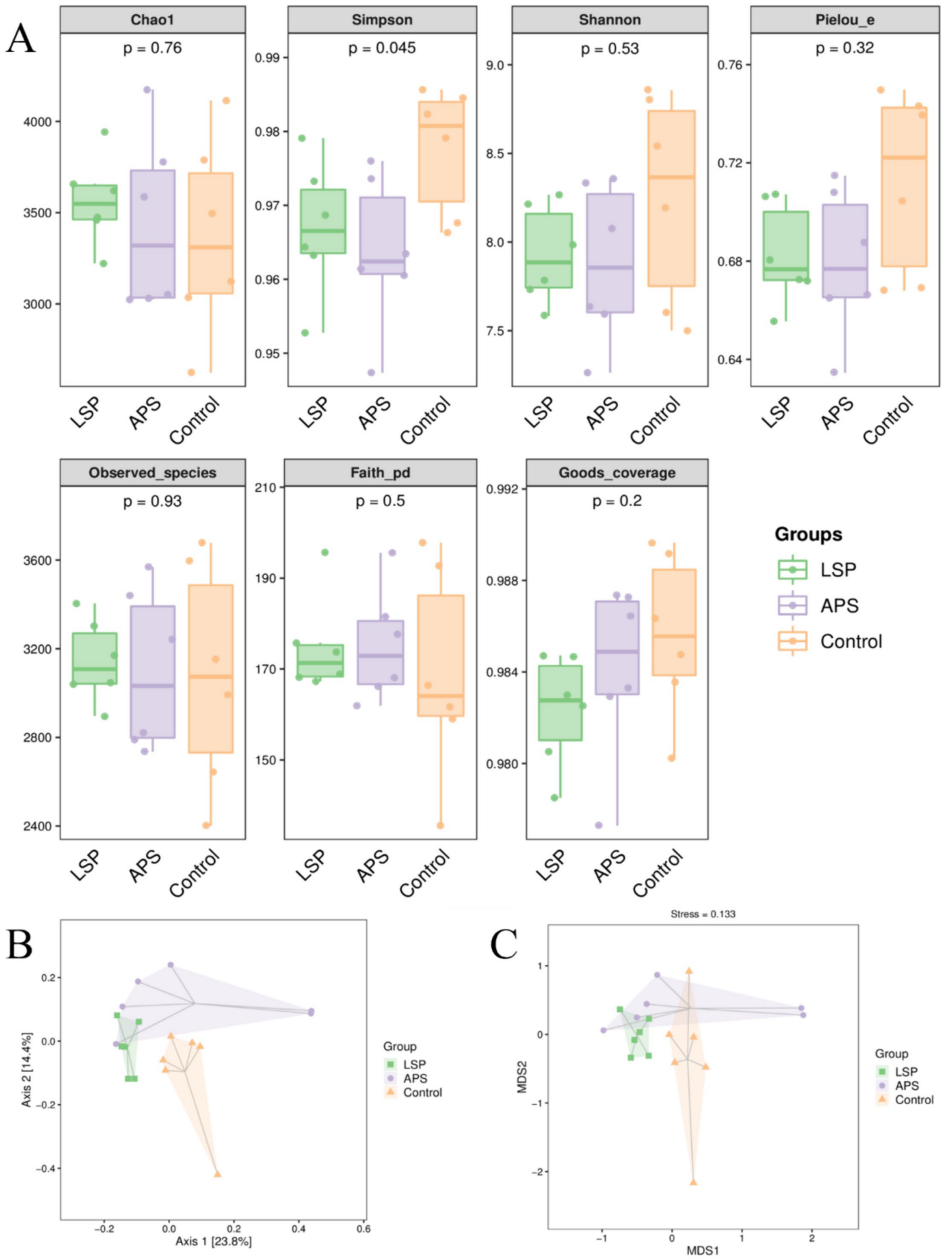
The impact of LSP on the diversity of intestinal microbiota **(A)** Grouped boxplot of alpha diversity indices. **(B)** Two-dimensional PCoA analysis plot. **(C)** Two-dimensional NMDS plot.

To acquire additional knowledge regarding the influence of LSP on the variety of gut microbiota, we utilized the Chao1 and Observed species indices to assess richness, and employing the Shannon and Simpson indices to evaluate diversity. Furthermore, we measured evolutionary diversity using the Faith PD index, assessed evenness through the Pielou e index, and evaluated coverage using the Goods coverage index. As shown in Figure 5A, the Chao1, Observed species, and Faith PD indices in the LSP group were higher than those in the control group, but the differences were not statistically significant (*p* > 0.05). The Simpson, Shannon, Pielou e, and Goods coverage indices in the LSP and APS group were lower than those in the control group, but the differences were not statistically significant (*p* > 0.05). The Principal Coordinates Analysis (PCoA) based on Bray-Curtis distances was conducted for the control, LSP, and APS groups. As depicted in Figure 5B, Axis 1 and Axis 2 eigenvalues accounted for 23.8 and 14.4% of the variation in sample community composition, respectively. When projected onto a two-dimensional plane within a 95% confidence interval, distinct separation between the LSP and control groups was observed, indicating disparities in intestinal microbiota between these two groups. Partial overlap was noted between the LSP and APS groups, suggesting that the dissimilarities in intestinal microbiota between these two groups were not statistically significant. To further investigate dissimilarities among groups, we conducted Non-Metric Multidimensional Scaling (NMDS) analysis, as depicted in Figure 5C, which revealed a substantial distance between the LSP and control groups, indicating their pronounced dissimilarity in terms of intestinal microbiota.

### 3.8 The impact of LSP on the microbial community composition at the phylum and genus levels

**Figure 6:**
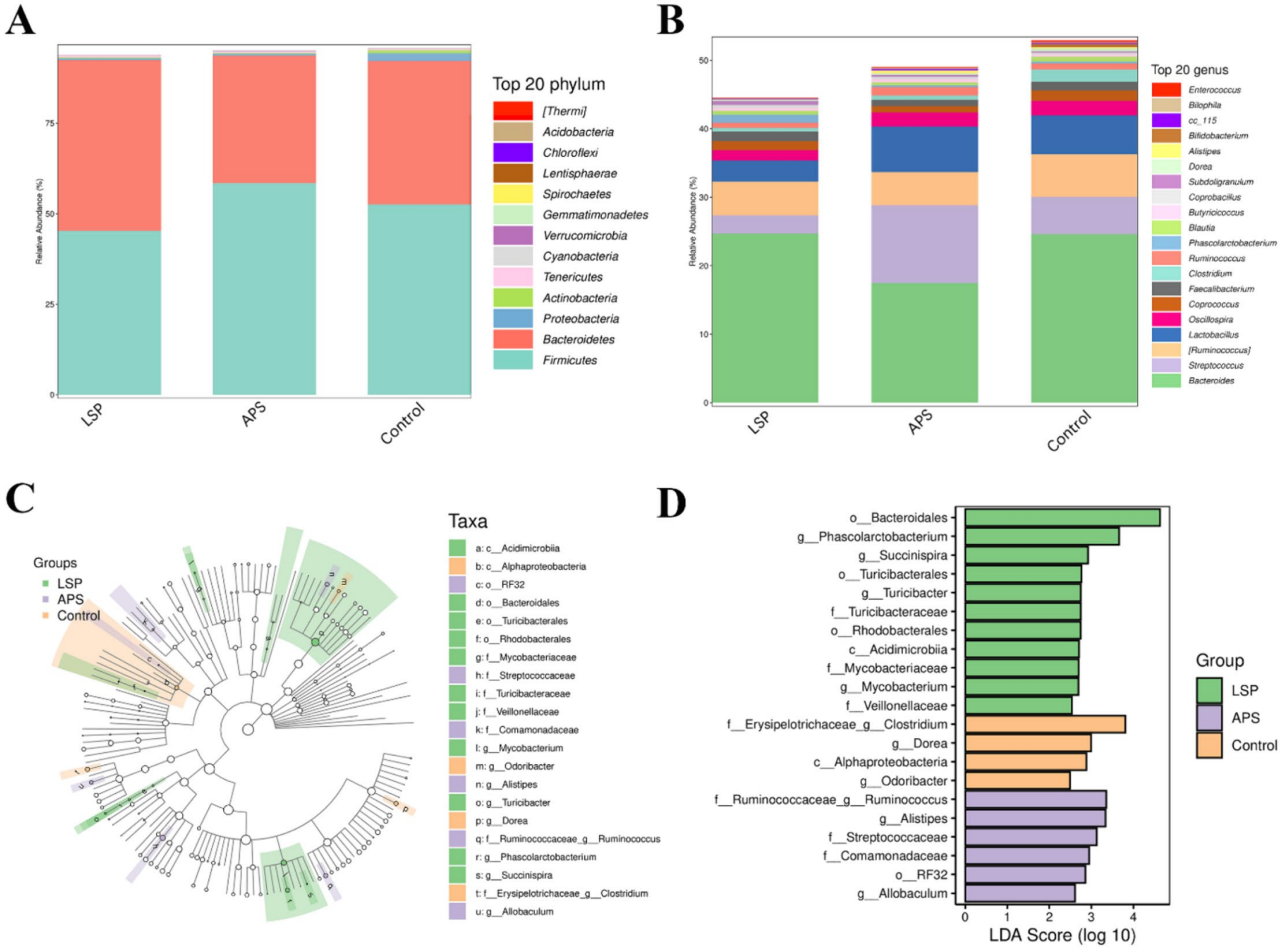
The influence of LSP on the composition of microbial communities. **(A)** bar plot of phylum-level species composition. **(B)** Bar plot of genus-level species composition. **(C)** Intergroup differences in taxonomic units based on classification tree. **(D)** Bar plot of LDA effect sizes for indicator species.

The composition of the broiler chicken intestinal microbiota at the phylum level is illustrated in Figure 6A, wherein Firmicutes and *Bacteroidota* emerge as the dominant phyla, collectively constituting over 75% of the microbial community. Notably, Firmicutes stands out as the predominant phylum. Comparatively, the LSP group exhibits an elevated abundance within the *Bacteroidota* phylum while displaying a reduced prevalence of Firmicutes. Conversely, the APS group demonstrates an increased predominance of Firmicutes accompanied by a diminished abundance of *Bacteroidota*. At the genus level, as depicted in Figure 6B, the prevailing genera comprise Bacteroides, Streptococcus, *Ruminococcus*, Lactobacillus, and *Faecalibacterium*; with Bacteroides being the most dominant genus. In comparison to the control group, the LSP group exhibited reduced abundances of Streptococcus, *Ruminococcus*, Lactobacillus, and *Oscillospira*; while there was an increase in the abundance of *Phascolarctobacterium*. The APS group displayed a decreased abundance of *Bacteroides* and increased abundances of *Streptococcus*, *Ruminococcus*, and *Lactobacillus*.

The LEfSe analysis results, as depicted in Figures 6C,D, revealed a total of 21 feature taxa, encompassing 2 at the class level, 4 at the order level, 7 at the family level, and 8 at the genus level. *Clostridium*, *Dorea*, *Alphaproteobacteria*, and *Odoribacter* were identified as characteristic taxa associated with the control group. *Ruminococcus*, *Alistipes*, *Streptococcaceae*, *Comamonadaceae RF32* and *Allobaculum* were found to be distinctive taxa linked to the APS group. *Bacteroidales Phascolarctobacterium Succinispira Turicibacterales Turicibacter gTuricibacter Turicibacteraceae Rhodobacterales Acidimicrobiia Mycobacteriaceae Mycobacterium* and *Veillonellaceae* were recognized as characteristic taxa associated with the LSP group. These findings further underscore that dietary supplementation with LSP induces alterations in microbial abundance across various taxonomic levels.

### 3.9 Effect of LSP on short-chain fatty acids

The levels of short-chain fatty acids (SCFAs) are presented in Table 4. Compared to the control group, the LSP group exhibited a significant increase in the concentrations of propionic acid, isobutyric acid, acetic acid, isovaleric acid, and caproic acid (*p* < 0.05). Furthermore, the addition of LSP to the diet significantly elevated the content of acetic acid in the cecum of broiler chickens when compared to both the control group and the APS group (*p* < 0.05).

**Table 4:** Content of different SCFAs in each group.

Items		Groups			
	Control	APS	LSP	SEM	*P*-value
Propionic acid	409.06^b^	564.58^a^	545.43^a^	46.91	<0.01
Isobutyric acid	42.76^b^	64.40^a^	62.09^a^	5.92	<0.01
Acetic acid	1605.80^b^	1645.07^b^	2186.21^a^	178.98	<0.01
Butyric acid	234.97^a^	254.84^a^	263.84^a^	33.49	0.692
Isovaleric acid	26.96^c^	53.42^a^	40.02^b^	5.28	<0.01
Valeric acid	55.98^a^	69.16^a^	54.68^a^	6.87	0.091
Caproic acid	0.69^b^	0.82^ab^	0.94^a^	0.11	0.098

Different lowercase letters on the data shoulder tags indicate significant differences (*P* < 0.05).

## 4 Discussion

The intestine is an open ecological system that directly interacts with the external environment. It serves as the largest digestive, absorptive, and immune organ within the animal body (22). Inhabiting the intestine is a vast array of microorganisms, which play a crucial role not only in host nutrient digestion and metabolism but also in organism development and their close associations with the host’s immune system and diseases. Simultaneously, intestinal microbes serve as essential factors in stimulating both the “intestinal mucosal immune system” and the “systemic immune system,” ensuring proper immune system functioning (23). As an immunomodulator derived from *Lagenaria siceraria*, LSP has been previously demonstrated to induce persistent high hemagglutination (HI) titers, antigen-specific IgA-NDV and IgG-NDV antibodies, splenic lymphocyte proliferation, and increased immune organ index to enhance immune response (14). However, there is a lack of research on the impact of incorporating LSP into broiler feed orally. This study aims to address this gap by examining the influence of dietary supplementation with LSP on growth performance, immune function, and intestinal microbial composition in broilers.

Growth performance and slaughter performance are crucial indicators for evaluating animal growth rate and achieving economic profitability. Enhanced growth performance implies reduced feed consumption and more significant weight gain in animal farming production. Slaughter performance is reflected in the processing of slaughtering, where lower losses during slaughter lead to higher production profits (24). Therefore, the poultry industry emphasizes key performance indicators such as ADWG, ADFI, F/G, and slaughter rate when processing broiler chickens. This study discovered that incorporating LSP into the feed significantly increased the average weight of broiler chickens, thereby improving both ADWG and F/G (Table 2). The effect of low dose LSP was more significant than that of medium and high dose LSP, which increased percentage of half-eviscerated yield, percentage of eviscerated yield, percentage of breast muscle, and percentage of leg muscle (Table 3). Indicating that LSP positively influences the growth and development as well as muscle quality of broiler chickens, thus enhancing both growth performance and slaughter performance.

Inflammatory cytokines have a crucial role in initiating immune responses and eliminating pathogens within the host (25). Previous research has shown that various feed additives can enhance the proliferation of T helper 1/T helper 2 (Th1/Th2) cells and stimulate the secretion of both pro-inflammatory and anti-inflammatory cytokines, thereby impacting the poultry immune system. IL-12 and IFN-*γ*, secreted by Th1 cells, are crucial for immune regulation (26). IL-12 promotes Th1 cell proliferation and IFN-γ production, which has been shown to be essential in resolving inflammation caused by primary *Salmonella* infection in chickens (27). Conversely, IL-4 induces Th2 cell proliferation while down regulating IL-12 production and inhibiting Th1 differentiation. Elevated levels of IL-5 contribute to lymphocyte activation, promoting bone marrow cell proliferation and differentiation as well as enhancing both cellular and humoral immunity levels (28). Secretory Immunoglobulin A (SIgA) is formed through Immunoglobulin A (IgA) aggregation within the intestinal mucosa; it plays a critical role in the intestinal mucosal immune response by selectively transferring to other mucosal tissues, establishing a broad immune response, stabilizing the mucosal barrier system, and preventing pathogenic microorganism invasion. Immunoglobulin IgG reflects virus-induced humoral immunity levels (29). In this study, supplementation of LSP significantly increased serum inflammatory cytokine levels (IL-4, IL-5, IL-12, IFN-*γ*), SIgA concentrations, and IgG titers (Figure 2). Suggesting that LSP can concurrently induce Th1/Th2 immunity while enhancing cellular, humoral, and intestinal mucosal immunity levels thereby reinforcing broiler chickens’ resistance against infectionand inflammation.

The thymus, spleen, and fabricius are pivotal immune organs in avian species. B lymphocytes undergo maturation within the fabricius, thereby acquiring a diverse repertoire of antibodies (30). In broiler chickens, the fabricius is more susceptible to exogenous feed influences compared to other organs (31). Wu et al. observed an increase in the relative weight of the thymus, spleen, and fabricius in broiler chickens when polysaccharide components were added to their daily diet (29). Our study observed no lesions or inflammatory infiltrations in various immune organs (thymus, spleen, fabricius) as well as cecal tonsils and ileum when supplemented with LSP. This indicates the safety profile of LSP supplementation. In addition, the indexes of thymus size, spleen weight and fabricius size of broilers supplemented with LSP were significantly increased, and the enhancement effect of low-dose LSP group was more significant. We propose that LSP might comprise compounds that facilitate the proliferation of advantageous microorganisms like lactic acid bacteria and spore-forming bacteria, thereby fostering immune organ development. Alternatively, it is possible that LSP contains prebiotic-like compounds (oligosaccharides) which facilitate development within broiler chicken’s immune organs (30).

The host’s digestion, intestinal development, nutrient absorption, and both innate and adaptive immune systems are regulated by the symbiotic relationship maintained with the gastrointestinal microbiota (32). The cecum, characterized by slow intestinal motility, low levels of antimicrobial substances, and weak alkaline pH, serves as a primary colonization site for microbiota (33). In this study, we investigated differences in the composition of microbial communities by supplementing LSP in the daily diet of broiler chickens. The results revealed an increased number of OTUs in the LSP group compared to the control group and APS group (Figure 4D). To further explore changes in the composition of the intestinal microbiota, this study conducted an analysis at the phylum and genus levels. At the phylum level, *Firmicutes* and *Bacteroidetes* dominated the intestinal microbiota, with a total abundance exceeding 75% (Figure 6A). These phyla play crucial roles in feed digestion and utilization, as they possess the ability to produce carbohydrate-active enzymes (CAZymes) responsible for breaking down complex indigestible polysaccharides, thereby promoting fiber degradation and carbohydrate utilization (34). At the genus level, the abundance of *Bacteroides* and *Phascolarctobacterium* increased in the LSP group (Figure 6B). *Bacteroides*, a group of Gram-negative, non-spore-forming, anaerobic, rod-shaped bacteria, ferment polysaccharides to produce volatile fatty acids (35). Additionally, *Phascolarctobacterium*, an obligate anaerobic, Gram-negative bacterium, produces short-chain fatty acids, including acetate and propionate (36). These findings align with previous research, suggesting that the bacterial community may become enriched and diversified through the degradation of LSP.

Short-chain fatty acids (SCFAs), including acetate, propionate, and butyrate, are the most prevalent and extensively investigated metabolites generated during bacterial fermentation (37). Research has demonstrated that SCFAs possess anti-inflammatory and immune-modulatory properties. Specifically, the fortification of the intestinal epithelium’s defensive role is heightened through the conversion of acetate into its corresponding salt structure. Propionate has been found to inhibit intestinal inflammation (38). Moreover, conversion of butyrate to its salt form not only stimulates mucin secretion in the animal intestine but also serves as a direct energy source for the intestinal mucosa, ensuring intestinal structural integrity and enhancing immune function (39). In this study, we observed a significant increase in the levels of propionic acid, isobutyric acid, acetic acid, isovaleric acid, and caproic acid in the LSP group compared to the control group (Table 4). This indicates that incorporating LSP into feed enhances the secretion of short-chain fatty acids within the gastrointestinal tract of broiler chickens.

## 5 Conclusion

In summary, LSP comprises monosaccharides such as galactose, rhamnose, arabinose, xylose, mannose, and glucose. Supplementation of LSP in feed induces alterations in the intestinal flora composition of broilers and enhances the secretion of short-chain fatty acids (propionic acid, isobutyric acid, acetic acid, isovaleric acid, and caproic acid), thereby promoting the development of immune organs like thymus, spleen and fabricius. These changes not only lead to increased levels of IL-4, IL-12, IgG and SIgA, but also positively impact parameters such as average daily weight gain (ADWG), average daily feed intake (ADFI), feed to gain ratio (F/G), percentage of half-eviscerated yield, percentage of eviscerated yield, percentage of breast muscle and percentage of leg muscle. Ultimately these improvements contribute to enhanced growth performance and immune function in broilers.

## Data Availability

The data presented in the study are deposition in the online repositories. The names of the repository/repositories and accession number(s) can be found below: https://www.ncbi.nlm.nih.gov/bioproject/PRJNA1164933.
